# A 6-month exercise intervention clinical trial in women: effects of physical activity on multi-omics biomarkers and health during the first wave of COVID-19 in Korea

**DOI:** 10.1186/s13102-024-00824-6

**Published:** 2024-01-29

**Authors:** JooYong Park, Jaemyung Kim, Jihyun Kang, Jaesung Choi, Ji-Eun Kim, Kyung-Joon Min, Seong-Woo Choi, Joo-Youn Cho, Miyoung Lee, Ji-Yeob Choi

**Affiliations:** 1 Department of Big Data Medical Convergence, Eulji University, Seongnam-Si, Gyeonggi-Do Korea; 2 Department of Biomedical Sciences Graduate School, Seoul National University, 103 Daehak-ro, Jongno-gu, Seoul, 03080 Korea; 3 School of Biological and Population Health Sciences, Oregon State University, Corvallis, OR USA; 4 Department of Clinical Pharmacology and Therapeutics, Seoul National University College of Medicine and Hospital, Seoul, Korea; 5 Institute of Health Policy and Management, Seoul National University Medical Research Center, Seoul, Korea; 6 DISPIS, Corporate, Namyangju-si, Gyeonggi-do Korea; 7 College of Physical Education and Sport Science, Kookmin University, 77 Jeongneung-ro, Seongbuk-gu, Seoul, 02707 Korea; 8 Cancer Research Institute, Seoul National University, Seoul, Korea

**Keywords:** SARS-CoV-2, Exercise, Biomarkers, Metabolome, Microbiota

## Abstract

**Background:**

Coronavirus disease 2019 (COVID-19) was first reported in December 2019 and the first case in Korea was confirmed on January 20, 2020. Due to the absence of therapeutic agents and vaccines, the Korean government implemented social distancing on February 29, 2020. This study aimed to examine the effect of physical activity (PA) on health through changes in multi-omics biomarkers with a 6-month of exercise intervention during the first wave of COVID-19 in Korea.

**Methods:**

Twenty-seven healthy middle-aged women were recruited and 14 subjects completed the exercise intervention. The mean age (± SD) was 46.3 (± 5.33) and the mean BMI (± SD) was 24.9 (± 3.88). A total of three blood and stool samples were collected at enrollment, after period 1, and after period 2 (3-month intervals). The amount of PA was measured with an accelerometer and by questionnaire. Clinical variables were used, including blood pressure, grip strength, flexibility, and blood glucose levels and lipid markers obtained from laboratory tests. The concentration of blood metabolites was measured by targeted metabolomics. Fecal microbiome data were obtained by 16 S rRNA gene amplicon sequencing.

**Results:**

During the second half period (period 2), Coronavirus disease 2019 occurred and spread out in Korea, and PA decreased compared with the first half period (period 1) (185.9 ± 168.73 min/week to 102.5 ± 82.30 min/week; *p* = 0.0101). Blood pressure, hemoglobin A1c (HbA1c), and low-density lipoprotein cholesterol (LDL-C) decreased in period 1 (*p* < 0.05) and tended to increase again during period 2 (*p* < 0.05). Forty metabolites were changed significantly during period 1 (FDR *p* < 0.05), and we found that 6 of them were correlated with changes in blood pressure, HbA1c, and LDL-C via network analysis.

**Conclusions:**

Our results may suggest that exercise improves health through changes in biomarkers at multi-omics levels. However, reduced PA due to COVID-19 can adversely affect health, emphasizing the necessity for sustained exercise and support for home-based fitness to maintain health.

**Trial Registration:**

The trial is retrospectively registered on ClinicalTrials.gov (NCT05927675; June 30, 2023).

**Supplementary Information:**

The online version contains supplementary material available at 10.1186/s13102-024-00824-6.

## Background

Coronavirus disease 2019 (COVID-19) is caused by severe acute respiratory syndrome coronavirus 2 (SARS-CoV-2) and was first reported in December 2019 in China [1]. The first case in Korea was confirmed on January 20, 2020, and the patient was imported from Wuhan, China [2]. The cumulative number of confirmed cases in Korea is over 27,995,000 (54.2%) by mid-December 2022.

On February 23, 2020, the alert level was raised orange to red due to the rapidly increasing number of confirmed cases in Korea, which resulted in the closure of all schools and one week of postponement of the start of school [2]. Due to the absence of therapeutic agents and vaccines, the only ways to prevent the spread of corona infection were quarantine, isolation, social distancing, or even lockdown, leading the Korean government implemented social distancing on February 29, 2020 [3].

Although social distancing or lockdown was introduced to prevent the spread of infection in many countries, it also significantly limited people’s daily activities due to restrictions on the use of public facilities. Decreased time spent in sports activities was observed in Italian children during the lockdown [4], weekly time physical activity (PA) also decreased in Spanish adults during national confinement [5, 6], and lower levels of PA were observed in United Kingdom adults with higher body mass index (BMI) during the lockdown [7]. Moreover, lower PA was reported post-COVID-19 among US adults who were active pre-COVID-19 [8], and decreased step counts were observed worldwide after the COVID-19 pandemic declaration [9]. As the beneficial health effects of PA are well known [10–12], the decreased PA caused by COVID-19 can lead to another health-related problem such as obesity, cardiovascular disease, or even mental health [13].

A number of epidemiological studies have shown the associations between PA and decreased risk of death and chronic diseases [10, 11]; however, biological processes and the underlying mechanisms for the benefit of PA are still unclear [14]. Recently, studies have been conducted to understand the benefits of PA more thoroughly way by using not only various anthropometric and blood biomarkers [15, 16] but also metabolites [17, 18] and microbiomes [19, 20]. PA induces changes in various objective measurements and clinical biomarkers in a health-promoting direction such as reducing levels of triglyceride, total cholesterol, blood glucose, and insulin, waist circumference, and body fat, while increasing HDL and muscle mass [15, 16]. The influence of PA on the metabolome shows a positive association with TCA cycle metabolites, including lactate, pyruvate, and ketones. In contrast, metabolites related to lipid metabolism, such as glycerophospholipids, sphingolipids, and bile acids, exhibit an inverse association with PA [17, 18]. Additionally, physical activity influences the microbiome, contributing to beneficial effects such as increased diversity [19, 20]. However, previous studies have been conducted at only one level, focusing on clinical variables, the metabolome, or the microbiome individually. There has been a lack of research that integrates various omic datasets, analyzing them collectively and confirming their relationships. This study was designed to examine the effect of PA on health through changes in various biomarkers including multi-omics level during exercise intervention.

1. To examine the effect of exercise intervention on clinical variables, metabolome, and microbiome.
2. To examine whether there is a washout effect after 3 months.

COVID-19 occurred during the intervention study, and a decrease in the amount of PA was observed thereafter. In this paper, we described the effects of reduced PA due to the outbreak of COVID-19 on health through various biomarkers at the multi-omics level.

## Methods

### Subjects and study design

Subjects were recruited from the women-only fitness center located in Gyeonggi-do. Eligibility to participate in the study included healthy women (without disease history including hypertension, dyslipidemia, type 2 diabetes, cardiovascular diseases, and cancers) aged 40 to 59 years, body mass index 18.5 or higher, planning or willing to participate in the exercise, and women with no physical limitations in exercise due to injuries or disorders of the musculoskeletal system. Presence of physical limitations in exercise was based on both consultation with fitness center experts and verbal statements from the subjects. After explaining the purpose and procedure of the study, written informed consent was obtained from the twenty-seven subjects who were willing to participate in the exercise intervention. This study was approved by the Institutional Review Board (IRB) of Seoul National University Hospital, Seoul, Korea (IRB No. 1812-129-997).

**Fig. 1:**
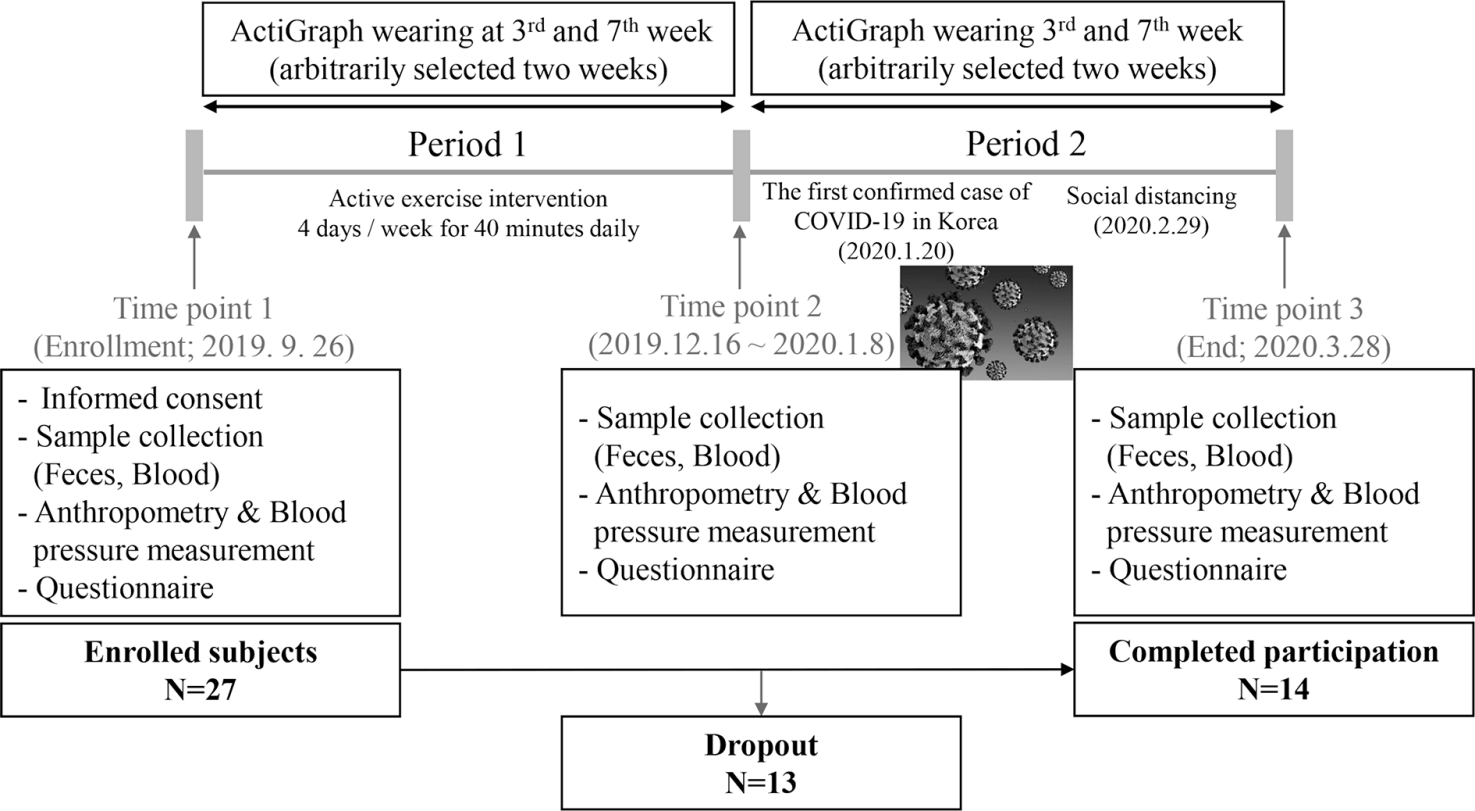
The study design and flow chart

This study was conducted with a longitudinal design with a loose intervention for 6 months. The research design referred to previous studies [21–26], and the protocol and detailed exercise program were constructed based on advice from fitness center experts who qualified as physical education instructors and exercise prescriptions. All subjects completed blood and fecal sample collections, measurements of blood pressure, grip strength, and flexibility, as well as responding to the provided questionnaires after enrollment (time point 1; September 26, 2019). Subsequently, subjects were involved in the aerobic exercise program at the women-only fitness center for 3 months (period 1). After period 1, subjects were recommended to exercise less than 150 min per week, and the women-only fitness center provided mainly a stretching exercise program (period 2) to examine the washout effect. All subjects completed the second and third sample collections, measurements, and questionnaires after period 1 (time point 2) and after period 2 (time point 3; March 28, 2020), as they had done at enrollment (Fig. 1).

During period 1, 10 subjects withdrew from study participation due to moving or illness. Three subjects who did not meet the criteria for wearing the accelerometer during period 2 were excluded. Therefore, 14 subjects were included in this study, and blood marker analysis and metabolomics analysis were performed in 13 subjects because one subject did not have a tertiary blood draw.

### Sample size calculation

We determined the minimum required sample size for the current study by utilizing estimates of *Akkermansia* from a previous study [23]. Our calculations indicated that a minimum of 12 subjects was necessary with power of 95% and a significance level of a = 0.05.

### Exercise intervention program

The exercise intervention program consisted of two distinct periods. During period 1, subjects participated in a moderate to vigorous intensity exercise program for 40 min a day, 4 days a week, at a women-only fitness center located in Gyeonggi-do. The exercise program was conducted in Tabata format, consisting of 3 min of exercise and 1 min and 30 s of rest. A moderate to vigorous intensity whole-body exercise program was performed for 3 min, targeting 70% of the maximum heart rate. Recovery was then induced for 1 min and 30 s, allowing the heart rate to return to resting levels. The exercise routine primarily consisted of full-body exercises using dumbbells, functional training using Total Suspension Training, and power exercises using gym sticks and medicine balls.

During period 2, a low-intensity static exercise program was implemented. The subjects exercised for 40 min a day, 4 days a week. The exercise program included static exercises such as Pilates and yoga, in addition to complex body strength training exercises. The maximum heart rate was maintained below 50%. The detailed exercise program consisted of stretching movements using foam rollers, bands, and massage balls, and balance movements using gym balls and balance pads.

### Physical activity measurements

Physical activity (PA) was investigated by a Korean version of the Global Physical Activity Questionnaire [27] at three time points (enrollment, after period 1, and after period 2) in person. Frequency and duration were investigated for both moderate-intensity leisure time PA (LTPA) and vigorous-intensity LTPA. The total time of LTPA was calculated as the sum of both vigorous and moderate LTPA (minutes per week).

In addition, PA was objectively measured by using a triaxial accelerometer (ActiGraph GT3X+; ActiGraph LLC, Pensacola, FL). During the examination, the device was fastened on their right waist within the midaxillary line using an elastic band. The participants were asked to wear an accelerometer for a minimum of 10 h/day and four out of 7 consecutive days. The objective measurements were implemented twice arbitrarily in period 1 and period 2, respectively (Fig. 1). The raw acceleration data were collected at a sampling rate of 30 Hz. Using ActiLife software (Version 6.12.1; ActiGraph LLC), the collected data were integrated into 60-sec epoch activity counts. For the wear time validation, the default Troiano 2007 algorithm was used [28]. The activity count cutoff point criteria used for PA intensity classification were as follows: sedentary behavior (0–99 counts/min), light (100–2,019 counts/min), moderate (2,020–5,998 counts/min), and vigorous (≥ 5,999 counts/min) intensity PA [18]. To evaluate participants’ PA time, we integrated the activity count, greater than or equal to 2,020 counts/min, into moderate-to-vigorous PA (MVPA). The average MVPA (minutes per week) measured during arbitrarily selected two weeks in each period represented the PA levels of period 1 and period 2, respectively.

### Blood sample collection

Blood samples were collected in the serum separation tubes (SSTs), EDTA tubes, and heparin tubes. Serum and plasma samples were obtained by centrifuging blood samples and were stored in a freezer at -80 °C until analysis [29]. Blood samples in the SST and EDTA tubes were used to obtain clinical variables through laboratory tests. Plasma samples obtained by heparin tube were used for targeted metabolomics analysis.

### Measurements of clinical variables

Systolic blood pressure (SBP) and diastolic blood pressure (DBP) (mmHg) were measured (Omron Corporation, Kyoto, Japan). Grip strength (kg) was measured for both hands and then averaged (TKK-5401). Flexibility (cm) was measured by the seated forward bend test. The concentrations of hemoglobin A1c (HbA1c) (mg/dL) (HLC-723G11 analyzer, TOSOH kabushiki kaisha, HPLC), glucose (mg/dL), total cholesterol (mg/dL), high-density lipoprotein cholesterol (HDL-C) (mg/dL), low-density lipoprotein cholesterol (LDL-C) (mg/dL), and triglyceride (mg/dL) (Cobas C701/702 autochemistry analyzer, Roche, enzyme method) were measured from whole blood in EDTA and serum samples in SST. The laboratory tests were conducted by GreenCross Pharma company (GC Pharma, Gyeonggi-do, Korea).

### Targeted metabolomics from blood sample

Plasma metabolite concentrations were measured by using the AbsoluteIDQ p 180 kit and the Bile acids kit (BIOCRATES Life Sciences AG, Innsbruck, Austria) with liquid chromatography mass spectrometry (LC–MS/MS) [30, 31]. Mass spectrometric analysis was conducted on an API 4000 QTRAP (Applied Biosystems/MDS Sciex, Foster City, CA, USA) equipped with an Agilent 1200 series high-performance liquid chromatography (HPLC) system (Agilent Technologies, Santa Clara, CA, USA). The AbsoluteIDQ p180 kit assay combines flow injection analysis and liquid chromatography, which can quantify 188 metabolites from six classes: amino acids, biogenic amines, glycerophospholipids, sphingomyelins, acylcarnitines, and hexose. The Bile acid kit assay can quantify 20 bile acid (cholic acid) metabolites. Metabolites were quantified and quality assessments were evaluated using MetIDQ software (Biocrates). The sample preparation and analysis were processed according to the manufacturers’ instructions. Metabolites were excluded when 30% of participants had below the limit of detection (LOD) in the two or three measurements. Finally, we used 142 metabolites, including 21 amino acids, 12 acylcarnitines, 70 phosphatidylcholines (PCs) (9 lyso-, 30 diacyl-, 31 acyl-alkyl-), 9 biogenic amines, 14 sphingomyelins (SM), 1 hexose, and 15 cholic acids. The remaining measurements below the LOD were imputed to half the LOD value of each metabolite.

### Fecal sample collection and 16s rRNA gene sequencing

Fecal samples were self-collected from subjects using commercial containers (Stool Nucleic Acid Collection and Preservation Tubes Cat. 45,630, Norgen BioTek Corp, Ontario, Canada) within 72 h after blood collection at the women-only fitness center and returning home. The collected samples were immediately shipped to the laboratory and stored in a freezer at -80 °C until analysis.

Fecal DNA was extracted and the V3 and V4 regions of 16s rRNA were sequenced. Detailed procedures were described in Supplementary Material 1. Sequence lengths less than 400 bp or over 500 bp were filtered out. Using CD-HIT-OUT, after removing low-quality reads and chimeric reads, species-level OTUs were assigned by clustering with more than 97% sequence similarity. Taxonomic assignment was performed with the organism information of the subject with the highest similarity by BLAST + (v2.9 0) on the reference DB (NCBI 16 S Microbial) [32].

### Statistical analysis

Total time spent PA per week, clinical variables, and α-diversity of the microbiome are described as the mean ± standard deviation. α-diversity (number of species, Chao1, Shannon, and Simpson indices) and relative abundance of microbiome were calculated by vegan package in R software (ver. 4.0.0).

Clinical variables, microbiome, and metabolites were compared between “Time point 1” and “Time point 2” to examine the effect of exercise intervention, and between “Time point 2” and “Time point 3” to examine the effect of reduced PA following the outbreak of COVID-19. For comparison between time points, paired t-test was used for clinical variables and Welch’s t-test was used for α-diversity. The Wilcoxon signed rank test was performed to compare the relative abundance of the microbiome or concentration of metabolites between time points, and multiple comparisons were adjusted to the false discovery rate (FDR). Bray-Curtis dissimilarity was calculated by the vegan package in R to measure β-diversity and to perform principal coordinate ordination analysis (PCoA) and permutational multivariate analysis of variance (PERMANOVA) in R. Spearman correlation coefficients were calculated between significantly changed clinical variables (*p* < 0.05), metabolites (FDR *p* < 0.05) and microbiomes (FDR *p* < 0.05). The network was visualized with the significant correlations between biomarkers (*p* < 0.05) by using Cytoscape software (ver.3.7.2).

## Results

Fourteen women completed participation in the exercise intervention program, which had been conducted for 6 months (Fig. 1). The characteristics of the subjects are shown in Table 1. The mean age (± SD) was 46.3 (± 5.33) and the mean BMI (± SD) was 24.9 (± 3.88). Most of the subjects were already involved in sufficient PA (≥ 150 min/week). During period 1, subjects’ objectively measured MVPA by ActiGraph, was average of 186 min per week. The level of MVPA was significantly reduced during period 2 (Table 2). During period 1, SBP, DBP, HbA1c, and LDL-C decreased significantly. These clinical variables increased again during period 2, while only HbA1c was significant (Table 2).

**Table 1:** Basic characteristics (Total N = 14)

Variables	N (%)
**Age, Mean ± SD (years)**	46.3 ± 5.33
40–44	5 (35.7)
45–49	6 (42.9)
50–54	1 (7.1)
55–59	2 (14.3)
**Body mass index, Mean ± SD (kg/m**^**2**^)	24.9 ± 3.88
< 25	6 (42.9)
≥ 25	6 (42.9)
Missing	2 (14.3)
**Education**	
≤Middle school	2 (14.3)
High school	6 (42.9)
≥College	6 (42.9)
**Income (**₩**10,000)**	
< 200	1 (7.1)
200–400	4 (28.6)
≥ 400	9 (64.3)
**Marital status**	
Living with spouse	12 (85.7)
Living alone	2 (14.3)
**Current occupation**	
Office	8 (57.1)
Unemployed/House wives	6 (42.9)
**Smoking**	
Never	10 (71.4)
Former	2 (14.3)
Current	1 (7.1)
Unknown	1 (7.1)
**Drinking alcohol**	
Never	1 (7.1)
Former	4 (28.6)
Current	7 (50.0)
Unknown	2 (14.3)
**Disease history**	
No	12 (85.7)
Yes	2 (14.3)
**Physical activity questionnaire, Mean ± SD (minutes/week)**	345.0 ± 298.01
< 150	2 (14.3)
150–300	6 (42.9)
≥ 300	6 (42.9)

**Table 2:** Changes of physical activity and clinical variables

	Time point 1	Period 1	Time point 2	Period 2	Time point 3
MVPA^1^, Mean ± SD (minutes/week)		185.9 ± 168.73^2^		102.5 ± 82.30	
Clinical variables					
SBP (mmHg)	135.9 ± 15.49^3^		127.6 ± 11.40		129.7 ± 15.62
DBP (mmHg)	89.9 ± 8.92^3^		82.0 ± 9.53		84.6 ± 8.80
Grip strength (kg)	28.0 ± 2.36		27.5 ± 2.38		27.4 ± 2.75
Flexibility (cm)	14.6 ± 11.48		13.9 ± 10.20		12.5 ± 9.93
Glucose level (mg/dL)	100.6 ± 13.60		104.0 ± 8.96		100.1 ± 15.33
HbA1c (mg/dL)	111.9 ± 8.28^3^		105.6 ± 9.51^4^		108.2 ± 8.26
Total cholesterol (mg/dL)	211.6 ± 31.77		207.1 ± 39.56		209.8 ± 39.01
Triglyceride (mg/dL)	168.2 ± 136.67		191.4 ± 233.12		111.1 ± 48.78
LDL-C (mg/dL)	134.0 ± 37.34^3^		118.5 ± 38.84		125.0 ± 36.14
HDL-C (mg/dL)	65.4 ± 20.14		62.9 ± 19.56		65.2 ± 18.69

Period 1: between “Time point 1” and “Time point 2”

Period 2: between “Time point 2” and “Time point 3”

Abbreviations: MVPA = moderate to vigorous physical activity; SBP = systolic blood pressure; DBP = diastolic blood pressure;

HbA1c = hemoglobin A1c; LDL-C = low-density lipoprotein cholesterol; HDL-C = high-density lipoprotein cholesterol

^1^ The average (minutes per week) MVPA was objectively measured by ActiGraph during arbitrarily selected two weeks in each period

^2^ Significant difference between “Period 1” and “Period 2” by paired t-test (*p* < 0.05)

^3^ Significant difference between “Time point 1” and “Time point 2” by paired t-test (*p* < 0.05)

^4^ Significant difference between “Time point 2” and “Time point 3” by paired t-test (*p* < 0.05)

**Fig. 2:**
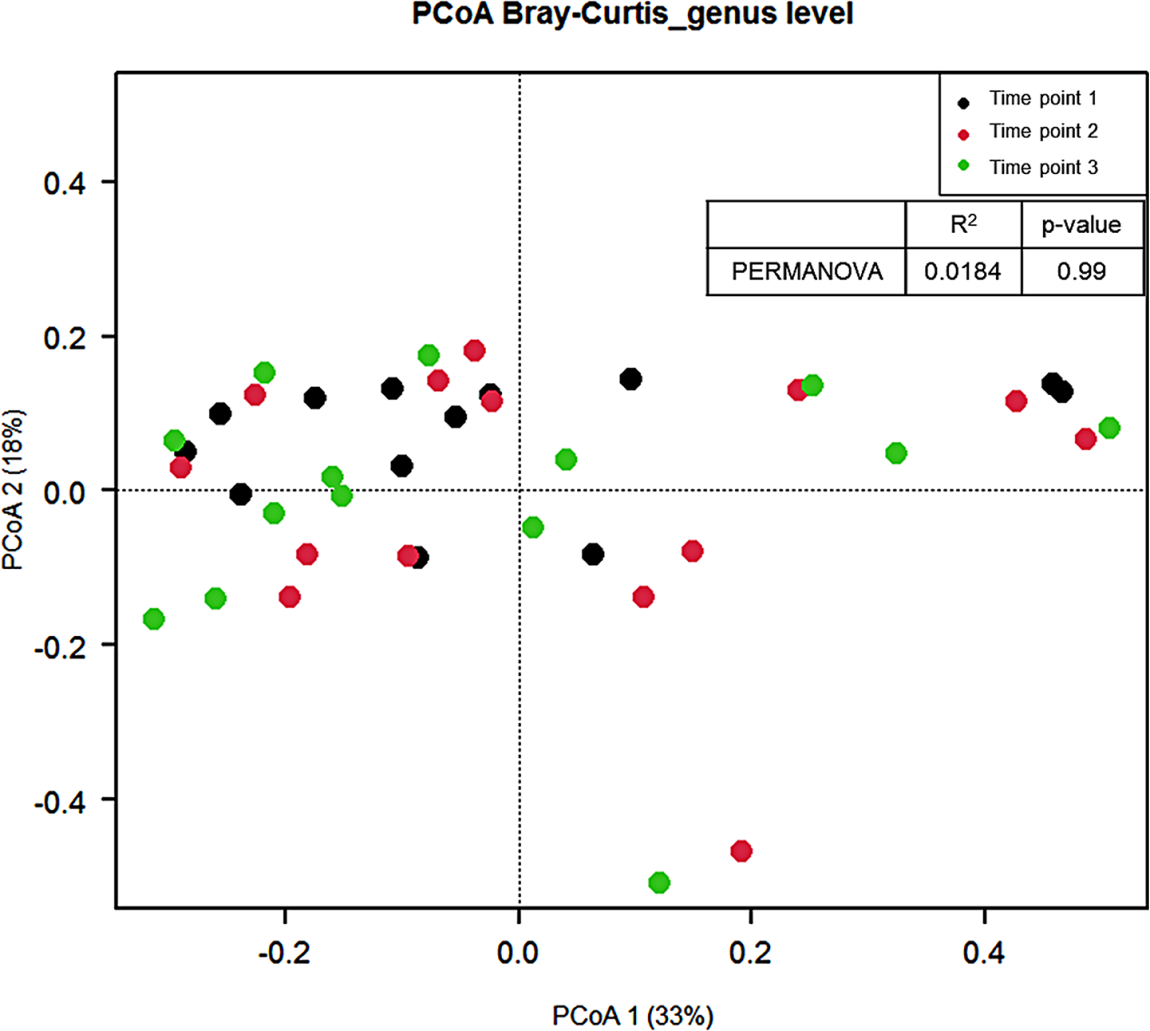
Principal coordinate ordination (PCoA) using Bray–Curtis dissimilarity between time points. Permutational multivariate analysis of variance (PERMANOVA) was performed to analyze beta diversity

Among the 142 metabolites, 71 metabolites were changed during period 1, and 37 metabolites were changed during period 2 (*p* < 0.05). After adjusting for multiple corrections, 40 metabolites during period 1 and 2 metabolites during period 2 were statistically significant. Overall, phosphatidylcholines, sphingomyelins, and bile acids were reduced during period 1. They were enhanced again during period 2, although most were not significant after adjusting for multiple comparisons (Table 3). Significant changes in the α-diversity indices during the intervention period (Table 4) or significant β-diversity between sample collection time points were not observed (Fig. 2). Differences in relative abundance between “time point” and “time point 2” were found for 6 microbial taxa at the genus level (*p* < 0.05) (Supplementary Fig. 1). Four microbial taxa at the genus level also showed differences between “time point 2” and “time point 3” (*p* < 0.05) (Supplementary Fig. 2). However, they were not statistically significant after adjusting for multiple comparisons.

**Table 3:** Changes in metabolites that were statistically significant during period 1 and period 2

Class	Period 1	p-value^1^	FDR-p	Period 2	p-value^1^	FDR-p
Biogenic amines	**Acetyl-ornithine **↓	**0.0081**	**0.0381**			
	Creatinine ↓	0.0215	0.0663	Creatinine ↑	0.0046	0.0732
	Putrescine ↑	0.0398	0.0991			
	**Serotonin** ↑	**0.0005**	**0.0058**	**Serotonin** ↓	**0.0005**	**0.0347**
	**Taurine** ↑	**0.0105**	**0.0436**	Taurine ↓	0.0024	0.0693
	**Total dimethyl-arginine** ↓	**0.0105**	**0.0436**	Total dimethyl-arginine ↑	0.0061	0.0788
Amino acids				Arginine ↑	0.0479	0.1836
	**Aspartate** ↑	**0.0025**	**0.0209**	**Aspartate** ↓	**0.0005**	**0.0347**
	**Glutamine** ↓	**0.0005**	**0.0058**	Glutamine ↑	0.0134	0.1059
	**Glutamate** ↑	**0.0118**	**0.0467**	Glutamate ↓	0.0105	0.0877
	Glycine ↓	0.0479	0.1152			
	Histidine ↓	0.0398	0.0991	Histidine ↑	0.0061	0.0788
	**Ornithine** ↓	**0.0002**	**0.0058**		0.0024	0.0693
	**Phenylalanine** ↓	**0.0061**	**0.0319**	Phenylalanine ↑	0.0266	0.1571
				Proline ↑	0.0175	0.1240
	**Tryptophan** ↓	**0.0046**	**0.0286**	Tryptophan ↑	0.0024	0.0693
Monosaccharides	Hexose ↓	0.0327	0.0893			
Acylcarnitines				C0 ↑	0.0012	0.0578
	**C16** ↓	**0.0012**	**0.0133**			
	C18:2 ↓	0.0215	0.0663			
Glycerophospholipids	**lysoPC a C16:0** ↓	**0.0017**	**0.0173**			
(Phophatidylcholines)	lysoPC a C16:1 ↓	0.0215	0.0663			
	lysoPC a C18:0 ↓	0.0266	0.0771			
	**lysoPC a C18:1** ↓	**0.0034**	**0.0221**			
	**lysoPC a C18:2** ↓	**0.0134**	**0.0489**			
	**lysoPC a C20:4** ↓	**0.0002**	**0.0058**	lysoPC a C20:4 ↑	0.0327	0.1682
				PC aa C28:1 ↑	0.0046	0.0732
	**PC aa C32:0** ↓	**0.0079**	**0.0381**			
				PC aa C32:3 ↑	0.0266	0.1571
	**PC aa C34:2** ↓	**0.0024**	**0.0209**			
	PC aa C36:2 ↓	0.0266	0.0771			
	**PC aa C36:4** ↓	**0.0005**	**0.0058**	PC aa C36:4 ↑	0.0191	0.1295
				PC aa C38:3 ↑	0.0171	0.1240
	**PC aa C38:4** ↓	**0.0034**	**0.0221**	PC aa C38:4 ↑	0.0081	0.0817
	**PC aa C38:6** ↓	**0.0097**	**0.0430**			
				PC aa C40:4 ↑	0.0360	0.1682
				PC aa C40:5 ↑	0.0426	0.1682
	**PC ae C32:1** ↓	**0.0134**	**0.0489**			
	PC ae C34:1 ↓	0.0392	0.0991			
	PC ae C36:2 ↓	0.0327	0.0893			
				PC ae C38:3 ↑	0.0330	0.1682
	**PC ae C38:4** ↓	**0.0005**	**0.0058**	PC ae C38:4 ↑	0.0398	0.1682
	PC ae C40:3 ↓	0.0398	0.0991	PC ae C40:3 ↑	0.0046	0.0732
	**PC ae C40:4** ↓	**0.0134**	**0.0489**	PC ae C40:4 ↑	0.0398	0.1682
	PC ae C40:6 ↓	0.0398	0.0991			
	PC ae C42:3 ↓	0.0277	0.0786			
	PC ae C44:6 ↓	0.0211	0.0663			
Sphingolipids	**SM (OH) C14:1** ↓	**0.0026**	**0.0209**	SM (OH) C14:1 ↑	0.0277	0.1571
(Sphingomyelins)	**SM (OH) C16:1** ↓	**0.0061**	**0.0319**			
	**SM (OH) C22:1** ↓	**0.0033**	**0.0221**	SM (OH) C22:1 ↑	0.0215	0.1387
	**SM (OH) C22:2** ↓	**0.0005**	**0.0058**	SM (OH) C22:2 ↑	0.0081	0.0817
				SM (OH) C24:1 ↑	0.0398	0.1682
	**SM C 16:0** ↓	**0.0002**	**0.0058**	SM C 16:0 ↑	0.0359	0.1682
	**SM C 16:1** ↓	**0.0002**	**0.0058**	SM C 16:1 ↑	0.0081	0.0817
	**SM C 18:0** ↓	**0.0026**	**0.0209**	SM C 18:0 ↑	0.0360	0.1682
	**SM C 18:1** ↓	**0.0005**	**0.0058**			
	SM C 20:2 ↓	0.0171	0.0607			
	**SM C 24:0** ↓	**0.0030**	**0.0221**	SM C 24:0 ↑	0.0424	0.1682
	**SM C 24:1** ↓	**0.0002**	**0.0058**			
Bile acids	**Cholic acid** ↓	**0.0108**	**0.0436**			
	**Chenodeoxycholic acid** ↓	**0.0061**	**0.0319**			
	Glycocholic acid ↓	0.0215	0.0663			
	Glycochenodeoxycholic acid ↓	0.0215	0.0663			
	**Glycodeoxycholic acid** ↓	**0.0086**	**0.0393**			
	Glycolithocholic acid ↓	0.0440	0.1078			
	**Glycoursodeoxycholic acid** ↓	**0.0005**	**0.0058**	Glycoursodeoxycholic acid ↑	0.0105	0.0877
	**Tauromuricholic acid** ↓	**0.0051**	**0.0300**	Tauromuricholic acid ↑	0.0408	0.1682
	**Tauroursodeoxycholic acid** ↓	**0.0063**	**0.0319**	Tauroursodeoxycholic acid ↑	0.0037	0.0732
	Ursodeoxycholic acid↓	0.0254	0.0766	Ursodeoxycholic acid ↑	0.0105	0.0877

Period 1: between “Time point 1” and “Time point 2”

Period 2: between “Time point 2” and “Time point 3”

↑: the concentration of metabolites increased during the period

↓: the concentration of metabolites decreased during the period

^1^Wilcoxon signed rank test (*p* < 0.05)

**Table 4:** Changes of α-diversity of microbiome

	Time point 1	Time point 2	Time point 3	p-value^1^	p-value ^2^
Number of species	108.3 ± 26.43	101.3 ± 22.91	95.2 ± 26.38	0.2065	0.2737
Chao1	113.6 ± 29.08	107.6 ± 25.81	102.2 ± 28.59	0.3839	0.3899
Shannon	2.70 ± 0.43	2.57 ± 0.42	2.47 ± 0.45	0.3501	0.2405
1/Simpson	8.41 ± 3.72	6.87 ± 2.93	6.99 ± 3.40	0.0818	0.7922

^1^ Paired Welch’s t-test between “Time point 1” and “Time point 2”

^2^ Paired Welch’s t-test between “Time point 2” and “Time point 3”

**Fig. 3:**
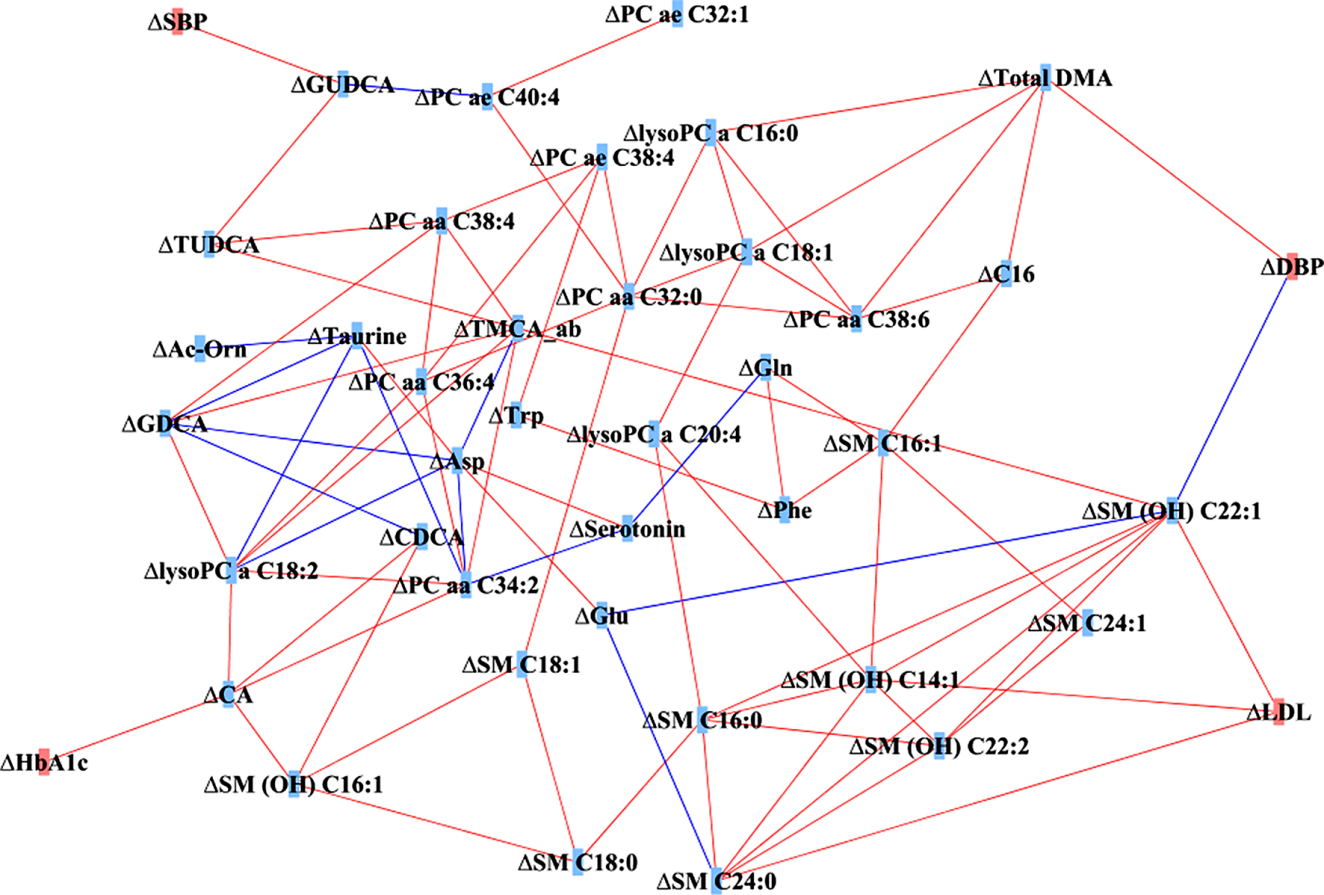
Network based on the Spearman correlation between changes in biomarkers during period 1 (between “Time point 1” and “Time point 2”); threshold: Spearman correlation coefficient *p* < 0.05 (42 nodes, 87 edges). Blue edges represents negative correlation, red edges represents positive correlation. Abbreviations: SBP = systolic blood pressure; DBP = diastolic blood pressure; HbA1c = hemoglobin A1c; LDL = low-density lipoprotein-cholesterol; Ac-Orn = acetyl-ornithine; Total DMA = total dimethyl-arginine; Asp = aspartate; Gln = glutamine; Glu = glutamate; Phe = phenylalanine; Trp = tryptophan; lysoPC = lyso-phosphatidylcholine; PC aa = diacyl phosphatidylcholine; PC ae = acyl-alkyl phosphatidylcholine; SM = sphingomyelin; CA = cholic acid; CDCA = chenodeoxycholic acid; GDCA = glycodeoxycholic acid; GUDCA = glycoursodeoxycholic acid; TUDCA = tauroursodeoxycholic acid; TMCA_ab = tauromuricholic acid (sum of alpha and beta)

Figure 3 shows a correlation-based network between changes in biomarkers during period 1. Any microbiome taxa were not included because there were no significantly different taxa between time points after controlling multiple comparisons. One of the sphingomyelins: SM (OH) C22:1 had the most edges (the highest degree), which means that it plays a central role in the network. SBP, DBP, HbA1c, and LDL-C, which were reduced clinical variables during period 1, were correlated with a few metabolites. The changes in SBP were positively correlated with changes in glycoursodeoxycholic acid (GUDCA), and changes in DBP were positively correlated with changes in total dimethylarginine (DMA) and negatively correlated with changes in sphingomyelin (SM (OH) C22:1). Changes in HbA1c were positively correlated with changes in cholic acid (CA). Changes in LDL-C were positively correlated with changes in 3 sphingomyelins: SM (OH) C22:1, SM (OH) C14:1, and SM C24:0 (Fig. 3).

## Discussion

This study aimed to examine changes in biomarkers, including clinical variables, metabolites, and the microbiome, during 6 months of exercise intervention that consists of two periods for 3 months each. During intervention period 2, the first confirmed case of COVID-19 in Korea was reported (on January 20, 2020), and as the number of confirmed cases continued to increase, social distancing was implemented on February 29, 2020. The objectively measured time of MVPA decreased in period 2 compared with period 1. Blood pressure, HbA1c, and LDL-C decreased during the exercise intervention of period 1, and they tended to increase again during period 2 when the amount of exercise decreased. Significant changes were observed in 40 metabolites during period 1, and we found that 6 of them were correlated with changes in blood pressure, HbA1c, and LDL-C.

We found that phosphatidylcholines, sphingomyelins, and bile acids were reduced in period 1, and they increased again in period 2, when the amount of exercise decreased due to COVID-19. These results were consistent with previous studies [33–35]. Phosphatidylcholines and sphingomyelins were positively associated with risk of cardiovascular diseases [36], and a biomarker of bile acid synthesis was associated with risk of metabolic syndromes [37]. Therefore, reduced phosphatidylcholines, sphingomyelins, and bile acids by exercise show the health benefit of exercise.

The α-diversity, which is known to be greater diversity that is generally beneficial for health, did not show significant changes during the intervention periods. Greater α-diversity was observed in the athletes than in healthy males in a previous cross-sectional study [38]; however, many longitudinal or randomized controlled trial (RCT) studies could not find any significant results for α-diversity involving exercise [21–26]. The maximum study period was 6 weeks among them. Recently, an RCT study conducted for 6 months in overweight or obese subjects showed that the vigorous intensity exercise group enhanced α-diversity compared with the control group [39]. These results suggest that significant changes in metabolites might be observed even in a short period of time, but long-term intensive exercise is required for a significant change in intestinal bacteria. Although our study was also conducted for 6 months, significant results were not obtained for the microbiome due to the reduced PA during period 2 of the COVID-19 outbreak.

In the correlation network between changes in biomarkers, we found links between clinical variables and metabolites. SBP was linked to GUDCA, and DBP was linked to total DMA and SM (OH) C22:1, which implies that decreasing blood pressure during exercise is closely related to decreasing bile acids and total DMA. Meanwhile, caution is needed in interpretation in that there is a negative correlation between sphingomyelin and DBP, which decreased during the exercise intervention period 1. We also found that HbA1c was linked to CA, which is one of the bile acids. A previous study showed that a biomarker of the bile acids synthesis was associated with a higher risk of metabolic syndromes [24]. Metabolic syndromes components include not only blood pressure but also glucose level or HbA1c. Bile acids synthesis occurs in the liver by cholesterol catabolism, and they activate a nuclear receptor: farnesoid X receptor, and a membrane receptor: G protein-coupled membrane receptor 5, to play a role in glucose and lipid metabolism [40–42]. Although the mechanisms regulating blood pressure or HbA1c by bile acids during exercise are not clear, previous studies have shown higher bile acids in patients with type 2 debates [43, 44] and positive associations of bile acids with blood pressure and fasting glucose [45]. Higher serum bile acids was also associated with the risk of coronary artery stenosis or plaques [46]; however, recent studies have suggested that GUDCA, which is a hydrophilic bile acid, may have the least toxicity or rather beneficial effects on the heart [47, 48]. Given that all types of bile acids were reduced together in our study, further studies are required to understand this inconsistency. The relationship between DMA and blood pressure was suggested by previous studies, which have shown that higher asymmetric DMA increased blood pressure, probably by raising systemic vascular resistance with a fall in cardiac output [49, 50].

LDL-C had three positive correlations with sphingomyelins: SM (OH) C22:1, SM (OH) C22:2, and SM (OH) C24:1. During period 1, both LDL-C and sphingomyelins were decreased. The detailed process of decreasing sphingomyelins via exercise is unclear; however, decreased ceramide with exercise was shown in a previous study [51]. Moreover, lower sphingomyelins were observed in the high weight loss group than in the low weight loss group by weight-loss intervention, including not only a reduction in energy intake but also the recommendation of PA [52]. Correlations between sphingomyelins and LDL-C were also shown [52]. Ceramides are important precursors for the biosynthesis of sphingolipids, and the ceramide transfer from the endoplasmic reticulum to the Golgi via ceramide transfer proteins is necessary for sphingomyelin synthesis [53]. During lipoprotein assembly by microsomal triglyceride transfer proteins, ceramides and sphingomyelins are incorporated into very low-density lipoproteins (VLDLs). After lipases hydrolyze triglycerides on VLDL, LDL can be formed [53]. These processes suggest that exercise-induced decreases in ceramide and sphingomyelin can lead to decreases in LDL-C. Further studies or experiments are needed to uncover the in-depth biological process regarding the relationship between LDL-C and sphingomyelins during exercise.

There are several limitations in this study. First, we intended to conduct a longitudinal design with a loose intervention. There were three repeated measurements, and subjects at baseline served as a control group when we examined the effect of exercise intervention. Similarly, when we examined the effect of reduced exercise, the subjects at the second measurement acted as a control group. Although not establishing a control group that does not receive any intervention, and having all subjects participate in the same schedule may be a limitation, it is not a significant one as a previous study conducted intervention studies without a separate control group [23]. The intervention during the second half period included a recommendation to exercise less than 150 min per week for subjects and a plan to provide a program focusing on stretching exercises in the women-only fitness center to examine the washout effect. However, it was practically impossible to exercise sufficiently due to COVID-19 that occurred during period 2. The amount of exercise significantly decreased in period 2 compared to period 1, and changes in biomarkers could be observed accordingly. Second, the target population was planned to be 60 to consider the dropout rate and increase statistical power, however, only 27 subjects were recruited. Although additional recruitment efforts were made in February 2020, exercise intervention could not proceed due to the spread of COVID-19. The dropout rate was close to 50%, much higher than expected. Ultimately, a small number of 13 subjects were included in the final analysis although the minimum required sample size was calculated to be 12 people. The fact that regression-based analyses accounting for dietary intake, BMI, and demographics information including smoking and drinking habits could not be conducted due to the limited number of subjects can also be a limitation. Nevertheless, we found statistically significant results of changes in clinical variables and metabolites, even their correlations. Last, significant results were not obtained in the microbiome. Although 10 genera were likely changes during the intervention, they were not significant after adjusting for multiple comparisons. As we described above, long-term and intensive exercise intervention might be required to find noteworthy results from the microbiome. Nevertheless, in this study design, we found that the amount of exercise decreased in the second half of the study in the aftermath of COVID-19 and we observed that HbA1c increased again during the second half. Metabolites belonging to the class of phospholipids, sphingolipids, and bile acids also decreased in the first half and increased again statistically significantly. This result suggests that reduced exercise due to COVID-19 can adversely affect health.

In this study, the effects of exercise on health were examined by using not only clinical variables that are available as indices of diseases but also metabolites and microbiomes expanded to the multi-omics level and their comprehensive changes. Furthermore, we showed the relationship between metabolites and clinical variables via a network. Social distancing or lockdown to contain the spread of COVID-19 are accompanied by restrictions on the use of many public facilities and curtailing people’s activities leading to unfavorable behavior changes [54, 55]. Reduced PA due to COVID-19 can have a negative impact on health including physiological biomarkers, sleep patterns, and even on mental health [8, 56, 57]. The COVID-19 pandemic continues as the new mutations of the coronavirus continue to occur, and the necessity for updating the global action plan for PA arises [58].

## Conclusions

The study highlights the positive effects of a 3-month exercise intervention on clinical variables including blood pressure, HbA1c, and LDL-C as well as their integrated relationships with several metabolites such as bile acids or sphingomyelins in middle-aged women. However, the emergence of the COVID-19 pandemic during the second half of the study resulted in reduced physical activity, causing the health improvements to reverse. This emphasizes the importance of continuous physical activity in maintaining the initial benefits. The research suggests promoting and supporting home-based exercise programs and virtual fitness platforms to mitigate the impact of external challenges on physical activity levels. By incorporating this approach into public health messaging and workplace wellness programs, individuals can sustain their physical activity levels, leading to improved cardiovascular health, glucose regulation, and overall well-being, regardless of external disruptions.

### Electronic supplementary material

Below is the link to the electronic supplementary material.

**Supplementary Material 1:** Protocol for the fecal 16s rRNA sequencing. **Supplementary Figure 1.** Significant difference of relative abundance between “Time point 1” and “Time point 2” in genus level (Wilcoxon signed rank test p < 0.05). **Supplementary Figure 2.** Significant difference of relative abundance between “Time point 2” and “Time point 3” in genus level (Wilcoxon signed rank test p < 0.05)

## Data Availability

The datasets generated and/or analysed during the current study are available in the Harvard Dataverse repository. 10.7910/DVN/98YY4E.

## Abbreviations

CA: Cholic acid
COVID-19: Coronavirus disease 2019
DBP: Diastolic blood pressure
DMA: Dimethylarginine
FDR: False discovery rate
GUDCA: Glycoursodeoxycholic acid
HbA1c: Hemoglobin A1c
HDL-C: High-density lipoprotein cholesterol
HPLC: High-performance liquid chromatography
IRB: Institutional Review Board
LC–MS/MS: Liquid chromatography mass spectrometry
LDL-C: Low-density lipoprotein cholesterol
LOD: Limit of detection
LTPA: Leisure time physical activity
MVPA: Moderate-to-vigorous physical activity
PA: Physical activity
PCoA: Principal coordinate ordination analysis
PCs: Phosphatidylcholines
PERMANOVA: Permutational multivariate analysis of variance
RCT: Randomized controlled trial
SARS-CoV-2: Severe acute respiratory syndrome coronavirus 2
SBP: Systolic blood pressure
SM: Sphingomyelins
SSTs: Serum separation tubes
VLDLs: Very low-density lipoproteins
