# Supplementary material for: A 6-month exercise intervention clinical trial in women: effects of physical activity on multi-omics biomarkers and health during the first wave of COVID-19 in Korea
Source: BMC Sports Sci Med Rehabil. 2024 Jan 29;16:30. doi: 10.1186/s13102-024-00824-6 (PMC10826212; doi:10.1186/s13102-024-00824-6)
Supplement: Supplementary file 1 — Supplementary Material 1: Protocol for the fecal 16s rRNA sequencing. Supplementary Figure 1. Significant difference of relative abundance between “Time point 1” and “Time point 2” in genus level (Wilcoxon signed rank test p < 0.05). Supplementary Figure 2. Significant difference of relative abundance between “Time point 2” and “Time point 3” in genus level (Wilcoxon signed rank test p < 0.05) [file 13102_2024_824_MOESM1_ESM.docx]

Supplementary Material 1. Protocol for the fecal 16s rRNA sequencing

DNA was extracted using a DNeasyPowerSoil Kit (Qiagen, Hilden, Germany) according to the manufacturer's instructions. The sequencing libraries were prepared according to the Illumina 16S Metagenomic Sequencing Library protocols to amplify the V3 and V4 regions.

The input gDNA (2 ng) was PCR amplified with 5x reaction buffer, 1 mM of dNTP mix, 500 nM each of the universal F/R PCR primer, and Herculase II fusion DNA polymerase (Agilent Technologies, Santa Clara, CA). The cycle condition for 1st PCR was 3 min at 95 °C for heat activation, and 25 cycles of 30 sec at 95 °C, 30 sec at 55 °C and 30 sec at 72 °C, followed by a 5-min final extension at 72 °C. The universal primer pair with Illumina adapter overhang sequences used for the first amplifications was as follows: V3-F: 5’-TCGTCGGCAGCGTCAGATGTGTATAAGAGACAGCCTACGGGNGGCWGCAG-3’, V4-R: 5’-GTCTCGTGGGCTCGGAGATGTGTATAAGAGACAGGACTACHVGGGTATCTAATCC-3’.

The 1st PCR product was purified with AMPure beads (Agencourt Bioscience, Beverly, MA). Following purification, the 2 µl of the 1st PCR product was PCR amplified for final library construction containing the index using NexteraXT Indexed Primer. The cycle conditions for the 2nd PCR were the same as those for the 1st PCR except for 10 cycles. The PCR product was purified with AMPure beads. The final purified product was then quantified using qPCR according to the qPCR Quantification Protocol Guide (KAPA Library Quantification kits for Illumina Sequecing platforms) and qualified using the TapeStation D1000 ScreenTape (Agilent Technologies, Waldbronn, Germany). Paired-end (2×300 bp) sequencing was performed by Macrogen using the MiSeq™ platform (Illumina, San Diego, USA).

Sequence lengths less than 400 bp or over 500 bp were filtered out. Using CD-HIT-OUT, after removing low-quality reads and chimeric reads, species-level OTUs were assigned by clustering with more than 97% sequence similarity. Taxonomic assignment was performed with the organism information of the subject with the highest similarity by BLAST + (v2.9 0) on the reference DB (NCBI 16S Microbial).

P=0.0249

P=0.0294

P=0.0094

P=0.0249

P=0.0175

P=0.0135

Supplementary Figure 1. Significant difference of relative abundance between “Time point 1” and “Time point 2” in genus level (Wilcoxon signed rank test p<0.05)
* None of them were statistically significant after adjusting multiple comparisons

P=0.0419

P=0.0353

P=0.0360

P=0.0203

Supplementary Figure 2. Significant difference of relative abundance between “Time point 2” and “Time point 3” in genus level (Wilcoxon signed rank test p<0.05)

* None of them were statistically significant after adjusting multiple comparisons
